# Identification of Sero-Diagnostic Antigens for the Early Diagnosis of Johne’s Disease using MAP Protein Microarrays

**DOI:** 10.1038/s41598-019-53973-x

**Published:** 2019-11-26

**Authors:** Lingling Li, John P. Bannantine, Joseph J. Campo, Arlo Randall, Yrjo T. Grohn, Megan A. Schilling, Robab Katani, Jessica Radzio-Basu, Laurel Easterling, Vivek Kapur

**Affiliations:** 1 Department of Veterinary and Biomedical Sciences, The Pennsylvania State University, University Park, PA United States of America; 2 Huck Institutes of Life Sciences, The Pennsylvania State University, University Park, PA United States of America; 3 National Animal Disease Center, USDA-ARS Ames, IA United States of America; 4 Antigen Discovery, Inc., Irvine, CA United States of America; 5 Department of Population Medicine and Diagnostic Sciences, Cornell University, Ithaca, NY United States of America; 6 Department of Animal Science, Pennsylvania State University, University Park, PA United States of America; 7 Applied Biological and Biosafety Research Laboratory, The Pennsylvania State University, University Park, PA United States of America

**Keywords:** Infectious-disease diagnostics, Proteomics

## Abstract

Considerable effort has been directed toward controlling Johne’s disease (JD), a chronic granulomatous intestinal inflammatory disease caused by *Mycobacterium avium* subsp. *paratuberculosis* (MAP) in cattle and other ruminants. However, progress in controlling the spread of MAP infection has been impeded by the lack of reliable diagnostic tests that can identify animals early in the infection process and help break the transmission chain. To identify reliable antigens for early diagnosis of MAP infection, we constructed a MAP protein array with 868 purified recombinant MAP proteins, and screened a total of 180 well-characterized serum samples from cows assigned to 4 groups based on previous serological and fecal test results: negative low exposure (NL, *n* = 30); negative high exposure (NH, *n* = 30); fecal-positive, ELISA-negative (F + E−, *n* = 60); and both fecal- and ELISA-positive (F + E+, *n* = 60). The analyses identified a total of 49 candidate antigens in the NH, F + E−, and F + E+ with reactivity compared with the NL group (p < 0.01), a majority of which have not been previously identified. While some of the antigens were identified as reactive in only one of the groups, others showed reactivity in multiple groups, including NH (*n* = 28), F + E− (*n* = 26), a*n*d F + E+ (*n* = 17) groups. Using combinations of top reactive antigens in each group, the results reveal sensitivities of 60.0%, 73.3%, and 81.7% in the NH, F + E−, and F + E+, respectively at 90% specificity, suggesting that early detection of infection in animals may be possible and enable better opportunities to reduce within herd transmission that may be otherwise missed by traditional serological assays that are biased towards more heavily infected animals. Together, the results suggest that several of the novel candidate antigens identified in this study, particularly those that were reactive in the NH and F + E− groups, have potential utility for the early sero-diagnosis of MAP infection.

## Introduction

*Mycobacterium avium* subspecies *paratuberculosis* (MAP) is the etiological agent of Johne’s disease (JD), a severe chronic intestinal inflammatory disease in ruminants and other animal species^1^. The disease is characterized by a long incubation period prior to manifestation of clinical signs, and subclinically infected animals represent a source of transmission to uninfected animals. MAP infection has a high prevalence in dairy herds in the US, as reported in 2007, at least 68% of US dairy farms were infected with MAP based on fecal and environmental sampling^2^, resulting in more than $200 million in annual losses to the dairy industry^3^. Efforts to control JD have been focused on reducing the transmission of MAP from infected cattle to uninfected young calves and removal of infected cattle (culling) from the herds^4^. Although it is well-recognized that the early identification of MAP infection is critical to prevent the spread of JD in herds, current diagnostic tests have a low sensitivity for detection of subclinical MAP infection.

Detection of MAP infection has been hampered in fecal tests for animals that show latent or intermittent shedding^5^, and serological tests, such as ELISA have shown low sensitivity in both low and moderate shedders, with only 26% testing positive with the currently available ELISA tests^6^. In order to develop more sensitive diagnostic tests, efforts have been focused on the discovery of novel antigens from MAP proteomic analyses, since the complete genome sequence was published^7^. A number of antigens have been characterized previously from cell wall associated proteins^8^; secreted MAP proteins^9^; proteins that respond to stress^10^; as well as MAP culture filtrate^11^ and cell extraction^12^. However, compared with the whole MAP proteome, only a small portion of proteins have been investigated, and for many studies, there were a limited number of well-characterized serum or milk samples used for evaluation.

To obviate the issues associated with this piecemeal approach to antigen discovery, we recently conducted a study using 180 well-characterized serum samples from cows to probe the whole proteome microarray from *Mycobacterium tuberculosis* (MTB)^13^. In the MTB array study, the cows were divided to 4 groups based on fecal (culture and PCR) and serum/milk ELISA tests: cows that were tested negative for both fecal and serum/milk ELISA and from JD-free farms (NL– Negative Low Exposure); those that were tested negative for both, but were from farms with existing JD (NH– Negative High Exposure); those that were fecal test positive and ELISA negative (F + E−); and those that were both fecal and ELISA tests positive (F + E+). With the NL group providing the baseline reference, a total of 47 MAP orthologs were identified from the NH, F + E−, and F + E+ groups as candidate antigens. The majority of candidate antigens, especially in the NH and F + E− groups, had not been previously recognized, indicating the MTB protein microarray approach had considerable utility for detection of MAP infection, especially during the early stages of MAP infection. However, there are limitations of the MTB array for MAP antigen discovery, including antigens for which there are no orthologs in the MTB proteome (unique MAP antigens), or MAP antigens that had low identity with their MTB orthologs^13^.

In order to overcome these limitations, we here report the development of a novel recombinant MAP protein array and the screening of sera from cows representing different stages of infection. The analyses identified several novel antigens that are recognized by cattle during various stages of MAP infection, including during the early stages that are currently difficult to diagnose using traditional approaches. Together, the results of our studies show that the use of MAP protein arrays has considerably expanded the pool of candidate antigens for the early detection of MAP-infected animals.

## Materials and Methods

All experiments and experimental protocols were performed in accordance with the relevant ethical animal care guidelines and regulations as per protocols approved by the Pennsylvania State University’s Institutional Animal Care and Use Committee (IACUC) protocol numbers 34625 and 43309.

### Bovine serum samples

The milk and serum samples were selected from the Johne’s Disease Integrated Program (JDIP, http://mycobacterialdiseases.org) diagnostic standards sample collection (samples were collected between August 2011 and February 2012). All samples have been previously analyzed using fecal culture and ELISA analysis for the detection of MAP (https://scholarsphere.psu.edu/concern/generic_works/hhm50ts37m).

The serum and milk were collected from cows housed California, Georgia, Minnesota, and Pennsylvania from 11 dairy farms with herd sizes ranging from 138 to 1400. The prevalence within each farm of JD ranged from 0–19.63%. All herds were tested for bovine TB and all were found negative^14^. As part of the integrated program, each cow was tested multiple times to assess the level of MAP present, including fecal culture, liquid culture (2 methods), acid fast staining, PCR detection, and ELISA, as described previously^14^. The 180 serum samples selected for this study, stratified into 4 groups: fecal and ELISA negative from JD-free herds (NL, n = 30), fecal and ELISA negative from infected herds (NH, n = 30), fecal positive and ELISA negative (F + E−, n = 60), and fecal positive and ELISA positive (F + E+, n = 60). The metadata associated with each of these cows can be accessed at: https://scholarsphere.psu.edu/concern/generic_works/hhm50ts37m. A summary of the farms and characteristics of the farms are in Supplemental Table 1. The samples were selected based on three different strata: the four different groups; ensuring representation of different dairy herds; and selecting the highest scores for PCR and ELISA tests to avoid selection of borderline or suspect positive samples.

### Preparation of recombinant proteins

Full-length coding sequences of each gene corresponding to the individual recombinant *MAP* proteins tagged with maltose binding protein (MBP) were amplified from the *MAP* K-10 genome with the restriction sites for *Xba*I and *Hind*III located in the 5′ and 3′ primers, respectively. The multi-gene fusion proteins were chemically synthesized and cloned in a similar manner. The ligation protocols were carried out overnight at 4 °C and transformed into *E. coli* DH5α and selected for on LB plates containing 0.10 mg/ml ampicillin. The ampicillin-resistant colonies were screen by PCR and DNA sequencing, as previously described^15^. The MBP-tagged recombinant proteins were overexpressed in culture using 0.3 mM isopropyl-B-d-thiogalactopyranoside (Sigma Chemical Company, St. Louis, MO). The cells were then harvested, re-suspended, put through one freeze-thaw cycle, and sonicated. The resulting crude extract was purified using amylose resin affinity chromatography, as previously described^14,15^. Purified proteins were eluted, and the most concentrated fractions were dialyzed with three exchanges of PBS at 4 °C. The purified protein was stored at −20 °C and assessed by a modified Lowry assay with a bovine serum albumin (BSA) standard. The recombinant proteins were also evaluated using the GelCode blue (Pierce Biotechnology Inc., Rockford IL)-stained SDS-PAGE gels.

### Microarray fabrication and probing

The MAP recombinant protein microarray fabrication and probing were performed at Antigen Discovery Inc. (ADI, Irvine, CA). A total of 868 purified MAP recombinant proteins were normalized to the concentration of 0.1–0.2 mg/ml before the array fabrication, with 64% of protein in the range of 0.1–0.2 mg/ml, 12% in the range of 0.05–0.09 mg/ml, and 24% were lower than 0.05 mg/ml. Normalized recombinant proteins and a reference protein MBP-LacZ were printed as single spots on a custom 8-pad nitrocellulose-coated Oncyte Avid slide (Grace Bio-Labs, Bend, OR) using an Omni Grid 100 microarray printer (Digilabs, Inc., Marlborough, MA) in 1 × 4 sub-array format, with each subarray comprising 18 × 18 spots^16^. The slides were rehydrated and blocked with Blocking Buffer (Main Manufacturing, Stanford, ME) for 30 minutes prior to incubating with diluted (1:200) serum samples at 4 °C overnight with gentle agitation. A biotinylated anti-bovine IgG secondary antibody (Jackson ImmunoResearch, West Grove, PA) and incubation with Surelight-P3 fluorochrome conjugated to streptavidin (Columbia Biosciences, Columbia, NY) were used to detect bound IgG antibodies. The dried slides were read with a Genepix 4300 A microarray scanner (Molecular Devices, San Diego, CA) that was calibrated daily. Fluorescence intensity for each spot was quantified using the GenePix Pro software as described previously^16^.

### Analysis of intensity data

Intensity data were analyzed using an automated pipeline developed at ADI and implemented in R (www.r-project.org) as described in detail in Li *et al*., 2017a. Briefly, spot measurements, normalizing for background intensities, were converted into a data matrix, quality checks were performed, and data was inspected for the presence of systematic effects and biases. Next, the dataset was log2 transformed, the median of the MAP-lacZ control spots were calculated, and the sample specific MBP-lacZ control medians subtracted. The antibody breadth score is the count of reactive antigens and they were compared using Poisson regression. Statistical analyses, both parametric and non-parametric tests, were performed to examine between-group differences. In the case of complex datasets, multivariate linear regression or linear mixed models with random effects for longitudinal data were performed. P-value adjustments for false discovery rates were also performed^17^.

Of the 868 recombinant MAP proteins spotted on the arrays, about 10% represented extracellular proteins, 40% membrane proteins, and 50% intracellular proteins as per predictions of subcellular localization^18^. A total of 661 proteins were recognized on the array as reactive proteins based on the intensities that measured 10% above the threshold, including 548 with a concentration ≥0.1 mg/ml and 113 with a protein concentration <0.1 mg/ml. Of the 207 proteins not reactive on the MAP array, 202 proteins were at a concentration <0.1 mg/ml, and only 5 were at a concentration of >0.1 mg/ml, and the association between protein concentration (≥0.1 vs <0.1 mg/ml) at slide fabrication and reactivity of spots was significant (p < 0.0001; Fisher’s exact and Chi-square tests), suggesting that non-reactive proteins in the MAP protein array were associated with a low concentration of the proteins as opposed to their true reactivity status.

Significantly reactive antigens were identified from 661 reactive proteins if an antigen met the following inclusion criteria: (1) Higher mean intensities in the NH, F + E−, and F + E+ groups when compared with that in the NL group using *t* test and Wilcoxon rank sum test with *p < *0.01. The p-value adjustment was made for multiple tests; for instance, for set 1, NL vs. NH; set 2, NL vs. F + E−; set 3, NL vs. F + E+, p < 0.0167 was considered as significant with Bonferroni’s correction as an adjustment; (2) Odds ratio (OR) in any one of the NH, F + E−, and F + E+ groups differed from that in the NL group (Supplementary Table S2); and, (3) AUC from the Receiver Operating Characteristic (ROC) curve was ≥0.7. Exceptions were made for antigens that only met criteria 1 and 2 but not 3 only if they were found to be reactive during our previous studies^16^.

### Statistical analyses

Logistic models were fitted using PROC LOGISTIC in SAS (version 9.2, 2009; SAS Institute Inc., Cary, NC) to examine the normalized intensities between the different antigens. Each antigen was included in the model and all models included basic information about the cow (lactation number, day-in-milk, herd size, etc.). Each non-baseline category was compared to the baseline category using the generalized logit function and the output included an odds ratio and 95% confidence interval for each antigen. Sensitivity and specificity for each antigen in the multiplex assays were generated using a Receiver Operating Characteristic (ROC) curve from the ROCR package in R (https://www.R-project.org/). Hierarchical clustering was performed using the pvclust package in R^19^.

## Results

### Screening antigens using a MAP protein microarray

**Figure 1:**
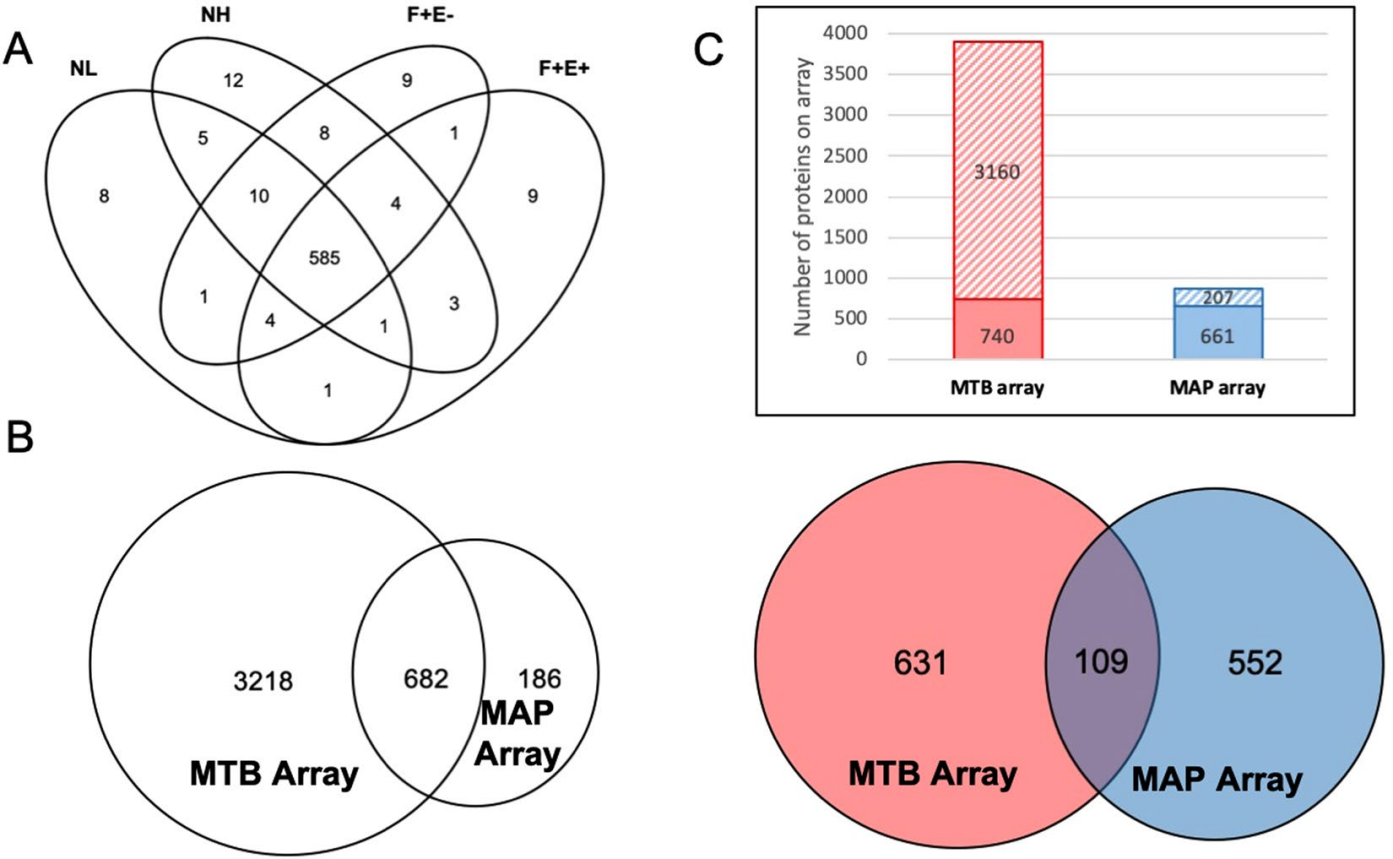
Reactive MAP proteins identified in this study and comparison of MAP and MTB protein array results. (**A**) Venn diagram at 10% threshold shows MAP antigen hit number distribution of all 4 groups: negative low exposure (NL), negative high exposure (NH), fecal positive & ELISA negative (F + E−), and fecal positive & ELISA positive (F + E+). (**B**) Orthologs between MAP and MTB arrays. Left circle represents MTB array containing 3,900 MTB proteins and right circle represents MAP array containing 868 proteins. The overlap region indicates orthologs between the two arrays. (**C**) Comparison of reactive proteins between MAP and MTB protein arrays. Top: number of reactive and non-reactive proteins in MTB and MAP arrays. Solid squares represent reactive proteins and twill squares represent non-reactive proteins. Bottom: red circle represents MTB reactive proteins and blue circle represents MAP reactive proteins. Overlap region represents reactive proteins identified by both arrays.

To identify strong antigens in MAP that could distinguish healthy dairy cows (NL) from those at early (NH), middle (F + E−) and late (F + E+) stages of Johne’s disease, we constructed a MAP protein array comprising 868 purified recombinant proteins. A total of 661 reactive MAP proteins were identified using this protein array on 180 serum samples from 11 dairy farms based on intensity thresholds described in the methods section. Of these proteins, 585 (88.5%) were recognized in all 4 stratified health status groups, and 38 (5.7%) were recognized only in one of the 4 groups (8 in NL, 12 in NH, 9 in F + E−, and 9 in F + E+), with the remaining 38 recognized by either 2 or 3 groups (Fig. 1A).

We next compared the reactivity data from the MAP array with our previous analysis of MAP seroreactive proteins using an MTB protein microarray corresponding to about 3,900 ORFs^16,20^. Of the 868 recombinant MAP proteins in this study, 682 had orthologs represented on the MTB array (Fig. 1B), of which a total 661 and 740 reactive proteins were recognized in MAP and MTB protein arrays (Fig. 1C, top figure), with 109 proteins shared between the two arrays (Fig. 1C, bottom figure). Of particular note, the majority of reactive proteins identified in this study were not recognized from the previous MTB protein array study, indicating that the MAP protein array has greatly expanded the pool of reactive proteins.

### Fourty-nine antigens are detected during early, middle and late stage disease

**Figure 2:**
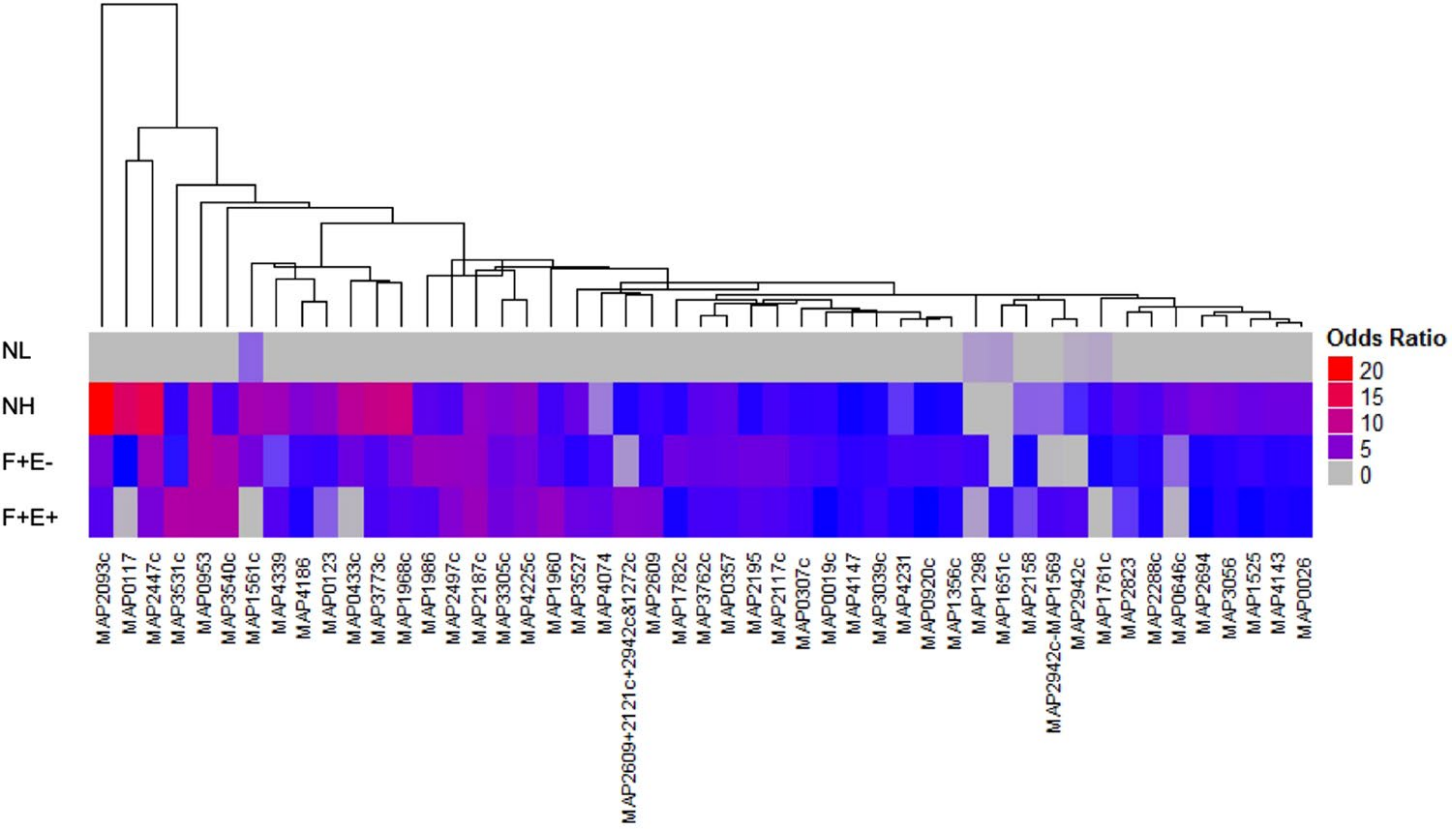
Patterns of serum reactivity to 49 MAP proteins with their odds ratios. Odd ratios differed significantly compared with that in the NL group. The heatmap shows reactivity from 4 groups to each of the 49 proteins. Each column represents one protein, odds ratios are visualized as a color spectrum. The heatmap was generated using the ComplexHeatmap package in R. The clustering was performed using the pvclust package with multiscale bootstrap resampling. Arguments passed to the pvclust command for the hierarchical clustering method (method.hclust) was “median” and for the distance method (method.dist) was “maximum”.

**Figure 3:**
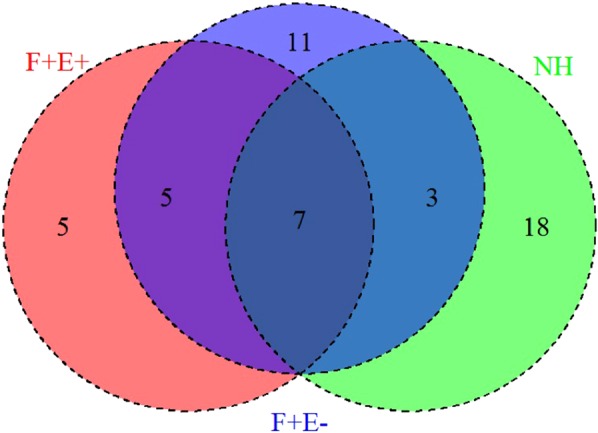
Venn diagram of 49 antigens identified in all three disease groups. The diagram shows antigen hit number distribution of all 3 groups: NH (green), F + E− (blue), and F + E+ (red). The three ellipses show the total number of hits from the 3 groups. Numbers indicate unique antigens identified in each group (non-overlapping areas) and shared antigens among groups (overlapping areas). Thirty four percent of antigens are shared among groups.

Based on the serological reactivity criteria described in the methods, a total of 49 antigens were identified across the NH, F + E−, and F + E+ groups. Serum reactivity to these 49 proteins were clustered based on their odds ratio values in each group (Fig. 2, Table S2). There were 18, 11, and 5 antigens identified only in the NH, F + E−, and F + E+ groups, respectively with the remaining antigens shared among 2 or 3 groups (Fig. 3). Functional descriptions of the 49 proteins are listed in Table S3. Most of the proteins (84%) have orthologs in MTB with an identity of 32% to 98% and 8 hypothetical proteins have no orthologs in MTB. The functional classification of these candidate antigens are found in the Supplemental Text, Section A.

### Early stage antigens

**Figure 4:**
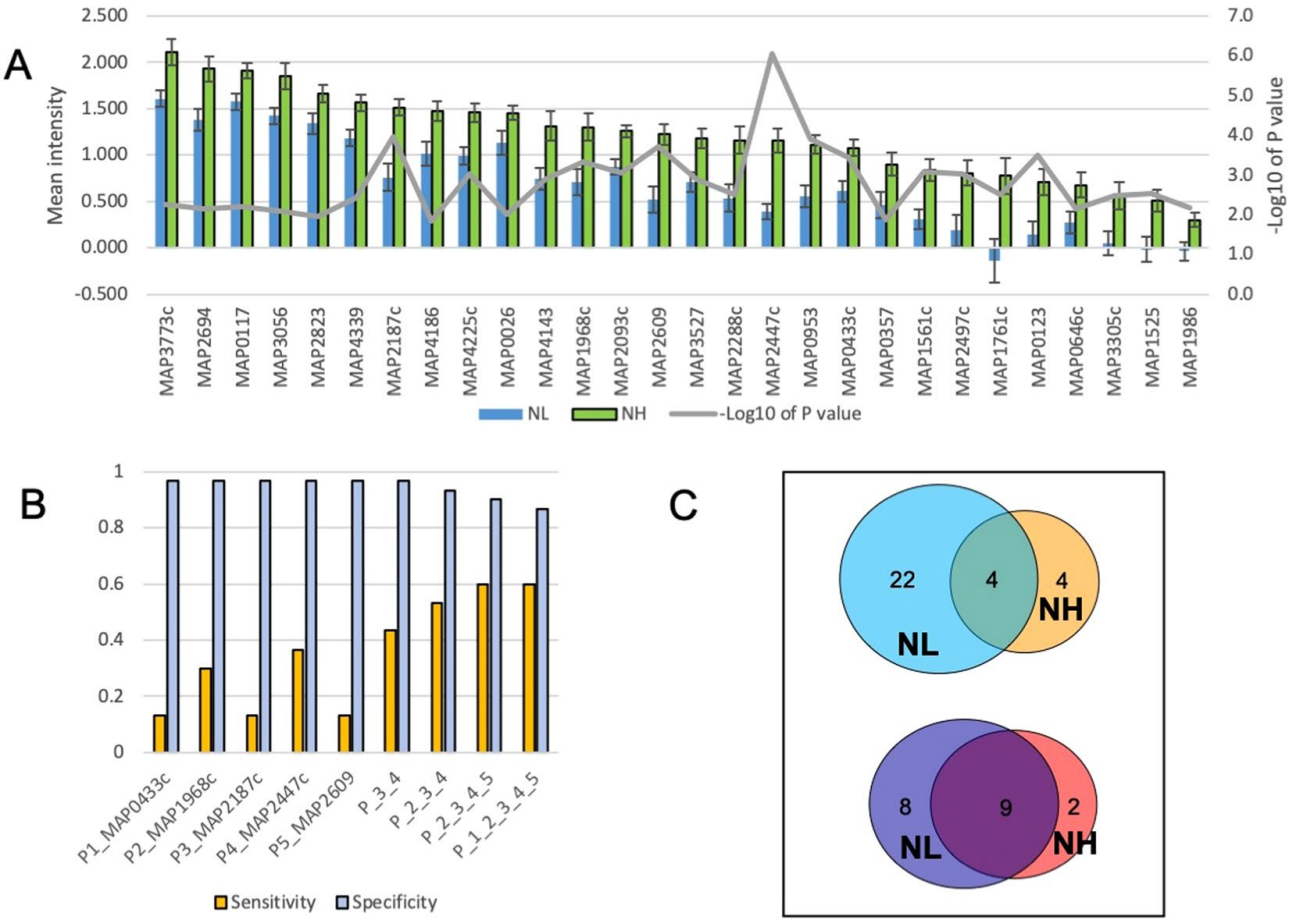
Antigens identified from the NH group. (**A**) The mean IgG reactivity to antigens was compared between the NH and NL mean intensity among 28 antigens. Corresponding p value (Wilcoxon test, −log10 of p value) to each antigen is shown in grey line on the secondary y axis. (**B**) Sensitivity of top 5 proteins at specificity 0.967 and sensitivity/specificity for combined antigens. (**C**) Different profiles of comparison of infected groups with NL and NH as a reference. Upper: number of significantly reactive proteins identified in F + E− group in comparison with NL (left circle) and NH (right). Bottom: number of significantly reactive proteins identified in F + E+ group in comparison with NL (left circle) and NH (right).

There was a total of 28 antigens identified in the NH group (Fig. 4A), including 18 unique to this group and 10 shared with others (3 with F + E−, and 7 with both F + E− and F + E+ groups). For the 18 antigens that were reactive only in the NH group, the mean intensities were higher than that for the F + E− and F + E+ groups, suggesting that these antigens are recognized only during early infection, but not as disease progresses. The functional classifications of these candidate antigens are found in the Supplemental Text, Section B.

Of the top 5 reactive antigens in the NH group based on the AUC, odds ratios, and predicted sensitivity and specificity, two antigens (MAP0433c and MAP1968) were reactive only in the NH group, whereas the other 3 (MAP 2447c, MAP2187c, and MAP2609) also showed reactivity in the other two groups (F + E− and F + E+).

The results show that at a specificity >0.95 (0.967) for the cutoff from the ROC curve, the sensitivity of individual antigens was low, ranging from 0.133 to 0.367. However, data show that when two, three and four antigens are combined, the sensitivity is increased from the highest individual sensitivity of 0.367 to 0.600 with a four antigen combination (Fig. 4B). However, there was no increase in sensitivity when 5 antigens are combined, but there is a loss of specificity for the 5-antigen combination (0.867) as compared with the 4 (0.900).

Similar to the results of our recent MTB protein array study^16^, the reactivity of the samples in the NL and NH groups were different from each other, suggesting that exposure to animals that may be shedding in the herd may increase sero-reactivity to certain MAP proteins. For instance, when the mean intensities in the NL and NH were used as a reference to compare the intensities in the F + E− and F + E+ groups, the number of antigens identified in these groups reduced from 26 (with NL as reference) to 8 (with NH as reference) in the F + E− groups and from 17 to 11 in the F + E+ group (Fig. 4C).

### Middle stage antigens

**Figure 5:**
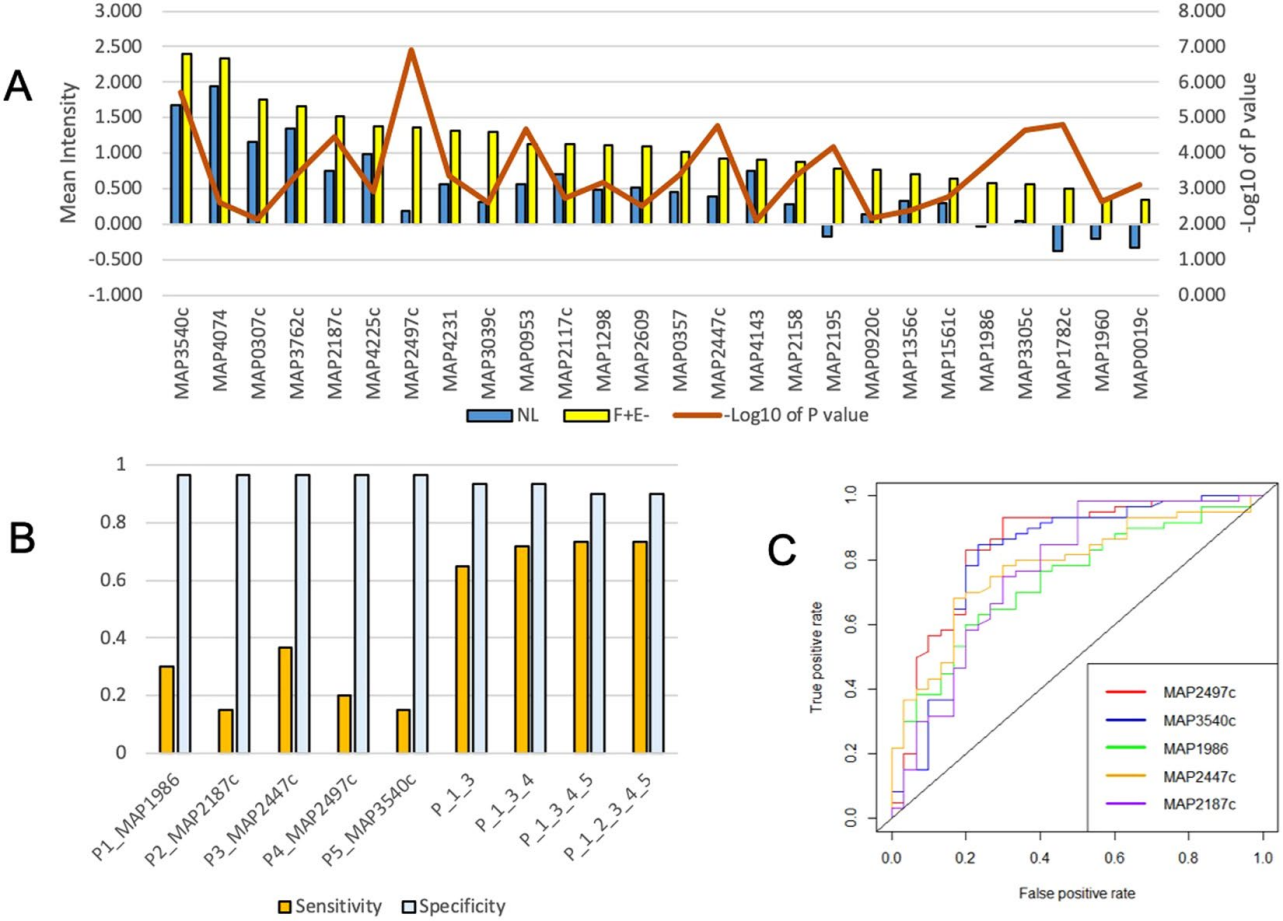
Antigens identified from the F + E− group. (**A**) The mean IgG reactivity to antigens (M ± SE) was compared between the F + E− and NL group. Corresponding p value (Wilcoxon test, −log10 of p value) to each antigen is shown in red line on the secondary y axis (**B**) Sensitivity of top 5 proteins at specificity 0.967 and sensitivity/specificity for combined antigens. (**C**) ROC curves for top 5 antigens in the F + E−.

There was a total of 26 antigens identified in the F + E− group, including 11 unique to this group and 15 shared with the NH, F + E+, or both (Supplementary Table S3, Fig. 5A). The functional classification of these candidate antigens are found in the Supplemental Text, Section C. A total of 12 antigens were excluded from the list of reactive antigens in the F + E− because of low mean intensity (<0.4; Supplementary Table S4). Among these, mean intensities in the NL group were all below 0, while mean intensities in the F + E− group ranged from below 0 to 0.24 with a median of 0.04. The most parsimonious hypothesis for the low observed intensity is likely to be a low concentration of spotted protein (all were <0.09 mg/ml, and four were <0.05 mg/ml). However, it is noteworthy that two of the significantly reactive antigens in the F + E−, MAP0920 and MAP1782c, showed strong reactivity despite having a low concentration (<0.05 mg/ml) of protein spotted on the array, suggesting that highly immunoreactive proteins may have strong signals even at very low concentration of antigens.

Both MAP2187c and MAP2447c were among the 5 most reactive antigens in the F + E− group and the NH group. The other three antigens were shared with other groups, MAP1986 shared with the NH, MAP3540 with the F + E+, and MAP2497c with both NH and F + E− group. Similar to the NH group, the sensitivities of individual antigens were low (0.15 to 0.367) but the combination of 4 antigens increased the sensitivity to 0.733 at a specificity of 0.9 (Fig. 5B). ROC curves of 5 top antigens are presented in Fig. 5C.

**Figure 6:**
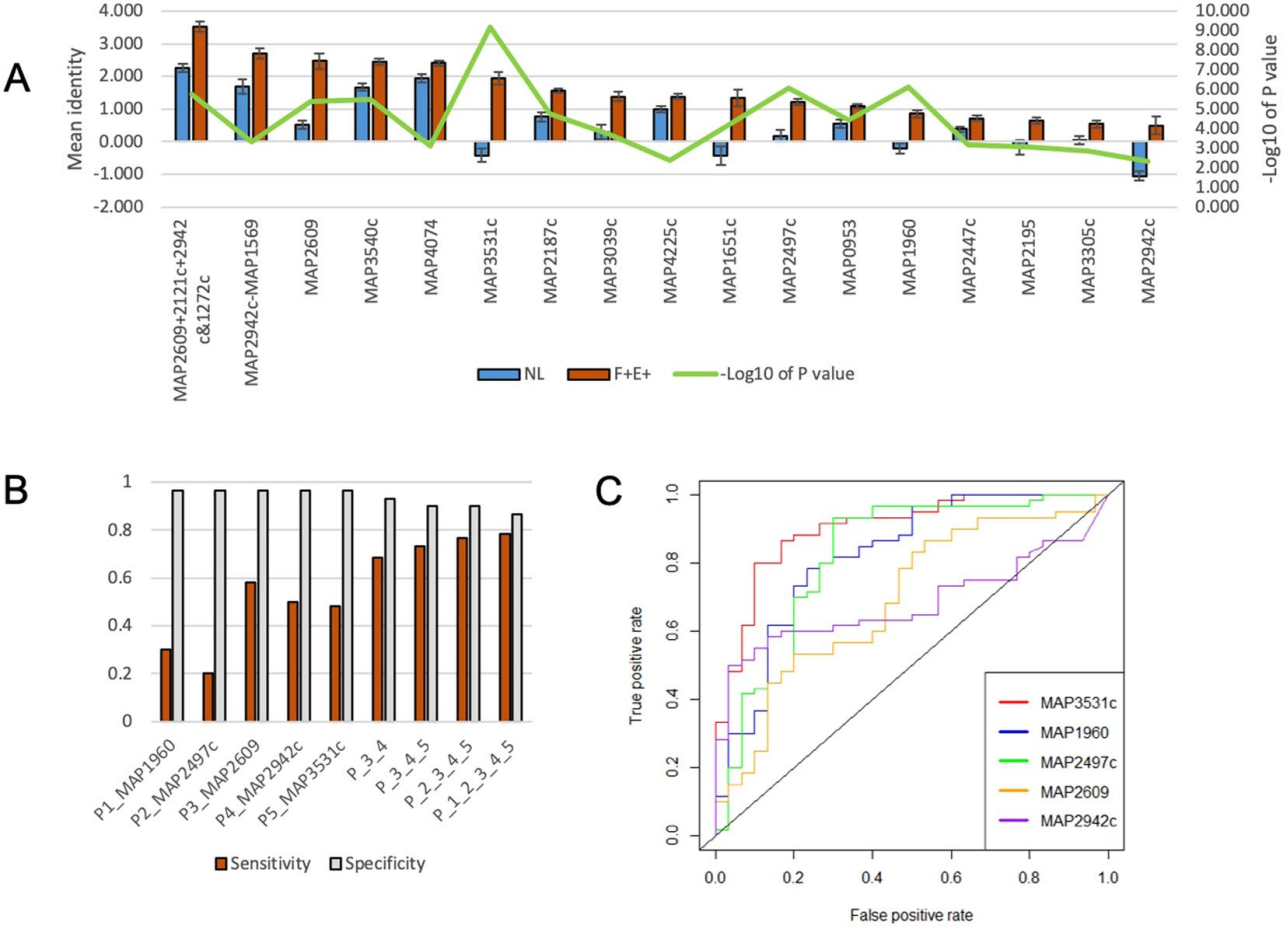
Antigens identified from the F + E+ group. (**A**) The mean intensities to antigens (M ± SE) was compared between the F + E+ and NL group. (**B**) Sensitivity of top 5 proteins at specificity 0.967 and sensitivity/specificity for the combined antigens. (**C**) ROC curves for the top 5 antigens in the F + E+.

There were a total 17 antigens identified as reactive in the F + E+ group, including 12 that were shared with other groups (5 with F + E− and 7 with both NH and F + E−), and the remaining 5 antigens were identified as reactive only with the F + E+ group (Supplementary Table S3, Fig. 6A). The functional classification of these candidate antigens are found in the Supplementary Text, Section D.

### Late stage antigens

Among the top 5 reactive antigens in the F + E+ group, MAP2942c and MAP3531c were identified only in this group, whereas MAP1960, MAP2497c and MAP2609 were shared with other groups. The sensitivity of individual antigens was higher than in the other two groups, ranging from 0.200 to 0.583. By combining the top 5 antigens, the sensitivity increased to 0.783 at a specificity of 0.867 (Fig. 6B). Considering all combinations of antigens, the results show that if MAP1960 is replaced by MAP1651c, predicted sensitivity increases to 0.817 with the 5- antigen combination, and specificity increases to 0.900, even though when considered individually, MAP1960 had a higher AUC value (Fig. 6C).

**Figure 7:**
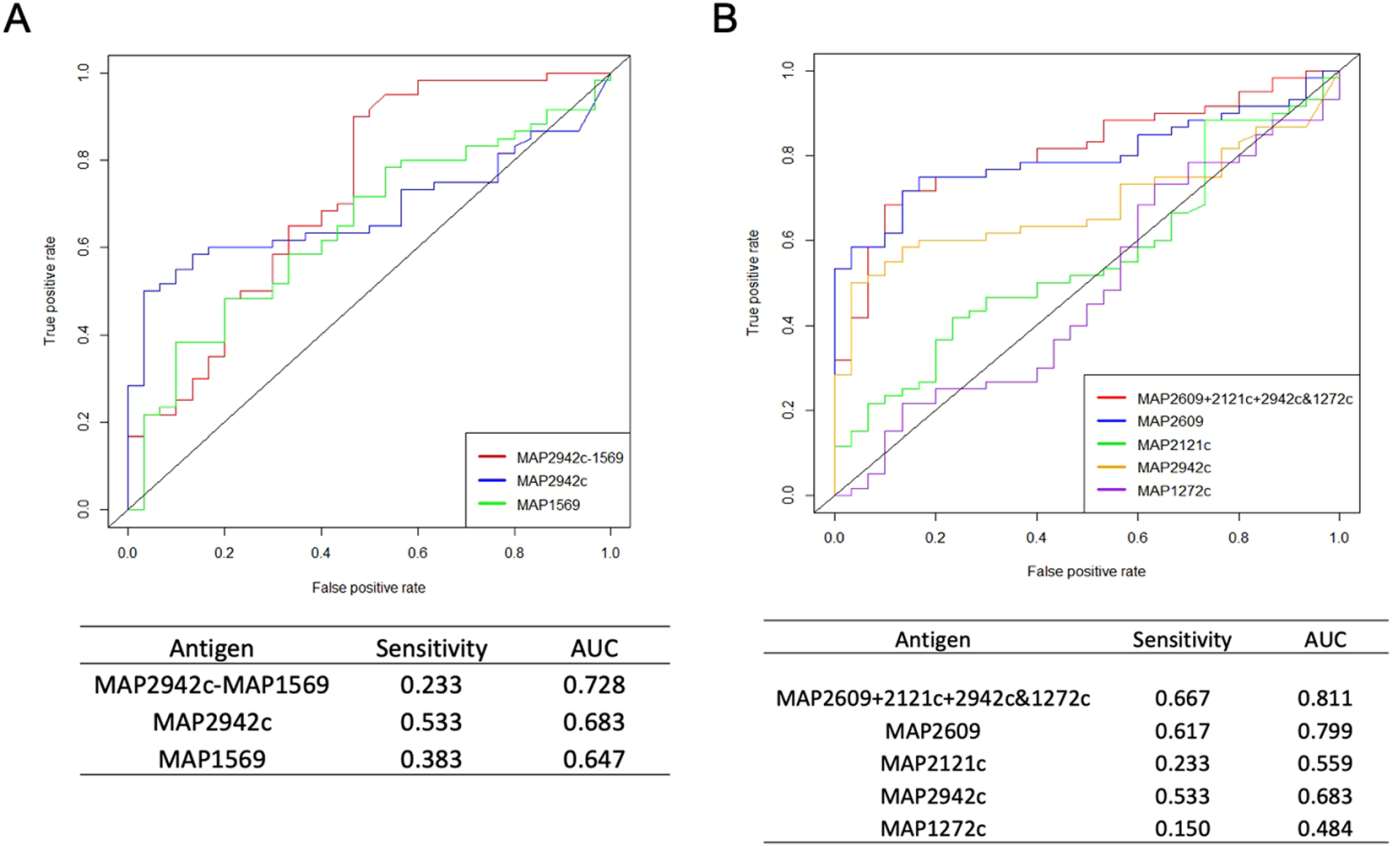
Comparison of fusion proteins with corresponding individual proteins. (**A**) Sensitivity (at specificity 0.9) and AUC of fusion protein MAP2942c-MAP1569 were compared with individual proteins MAP2942c and MAP2569. (**B**) Sensitivity and AUC of fusion protein MAP2609 + 2121c + 2942c&1272c were compared with 4 corresponding individual proteins.

The multi-protein fusions MAP2942c-MAP1569 and MAP2609 + 2121c + 2942c + 1272c were identified among the significantly reactive antigens in the F + E+ group, each of which has previously been identified as reactive when used as individual antigens in previous studies^21–24^. Compared with their corresponding individual proteins for sensitivity and AUC values, the fusion protein MAP2942c-MAP1569 had a higher AUC compared with each of the individual proteins (Fig. 7A), but the sensitivity (at 0.9 specificity) was lower than for either MAP2942c (0.233 vs 0.533) or MAP1569 (0.233 vs 0.383) alone. Of the other tetra-fusion protein MAP2609 + 2121c + 2942c + 1272c, two antigens (MAP2609 and MAP2942c) were identified as reactive in the current study, while MAP2121c and MAP1272c were not (Fig. 7B). The tetra-fusion showed sensitivity and an AUC value slightly higher than MAP2609, however, it had a much higher background intensity compared to MAP2609 (mean intensity 2.26 in fusion vs 0.52 in MAP2609 in the NL group, p < 0.0001). Consequently, the ratio of mean intensity (F + E+/NL) for the fusion protein was lower than in MAP2609, (4.8 vs 1.6). Based on the above preliminary analyses, the fusion proteins represented in the current investigation did not show obvious advantages over individual proteins, perhaps due to the higher background that will require further optimization, and hence were not included in the top 5 antigens in the F + E+ group.

## Discussion

The aim of this study was to identify reactive antigens at different stages of Johne’s disease. We demonstrate that there is a dynamic humoral immune response as Johne’s disease progresses in the dairy cow. Using a novel MAP protein array and a well-characterized serum sample bank, we identified 661 seroreactive MAP antigens, a majority of which did not show significantly increased reactivity in the NH, F + E−, and F + E+ groups as compared with that of the NL group. These results, consistent with data from previous studies^16^, suggest that if animals in the NL group are most likely to be considered as truly negative, there is significant “background” or cross-reactivity of MAP proteins with serum from cattle that are presumed to be unexposed or uninfected with MAP. While the provenance of this “background” or “cross-reactivity” is unknown and might include exposure to environmental or other mycobacteria (including other members of the ubiquitous *Mycobacterium avium* complex), or a lack of specificity of the assay format, or some such combinations of these or other factors, the results highlight the importance of the need for rigorous and robust statistical analyses along with well-stratified sample sets for the identification of antigens of sero-diagnostic importance for MAP in cattle.

The lack of a gold standard diagnostic test has been a major limitation for the development of sensitive diagnostic tests for MAP infection. In particular, the reliance on fecal assays, whether PCR- or culture-based, that are limited in sensitivity because of both biological (for eg., due to intermittent shedding during early infection) and technical reasons (for eg., loss of nucleic acid or inactivation of MAP during extraction or decontamination) represents a real conundrum since they may not accurately reflect the infection status of the animal. To obviate some of these challenges, Bayesian approaches have been developed^25^; however, to our knowledge, these have not been applied to extant commercial serological assays, perhaps, in-part, due to the nature of the crude antigen preparation that has a greater likelihood to cross-react with environmental bacteria and hence cut-off values are established to favor specificity thereby decreasing sensitivity.

Similar to what was observed with the earlier MTB arrays, serological reactivity to antigens in the NH group significantly differed from the reactivity in the NL group. Cows in both groups were negative for MAP at the time of sample collection based on the observed negative ELISA assays and very low apparent infection rates as determine by fecal tests, however, the farms included in the NH group had previously infected animals. To further define the apparent increase in sero-reactivity among negative cows, we compared the two different ELISA methods used in this study, ELISA IDEXX and ParaChek, using serum from negative animals in uninfected (n = 195 in NL) and infected farms (n = 216 in NH), as well as with infected cows from infected farms (n = 159 in F + E− and n = 98 in F + E+; Supplemental Table S2, Fig. 1B). With the ParaChek ELISA, a significantly higher OD value was noted for the F + E+ group and no difference was observed between the NH and F + E− groups compared to the NL (p > 0.05). With the IDEXX ELISA, the mean OD values were significantly higher in the F + E+ group compared to the other 3 groups as well as in the NH and F + E− groups compared to the NL group (p < 0.001). However, importantly, there was no significant difference in reactivity between the NH and F + E− group. In contrast, using the NH as a reference, as compared to the NL group as a reference, greatly reduces the antigens identified in the F + E− and F + E+ groups, which is consistent with the observation with IDEXX ELISA that mean OD in the F + E− group was significantly higher than that in the NL, but not in the NH group (Supplemental Table S2, Fig. 2).

To begin to identify factors that may contribute to the increased sero-reactivity in the NH group, fecal shedding patterns of animals in the NL farms (Farm A and G) were compared with infected animals (Supplemental Table S2, Fig. 2A). In brief, there are two primary observed differences between the NL and the infected farms. First, the detection rate for positive fecal samples in uninfected Farms A and G was low, ranging from 2.5% to 7.7%, as compared with a fecal positivity range of 25.0% to 98.3% in infected farms (P < 0.05). Second, the observed levels of MAP fecal shedding were low in cows with positive fecal tests from the NL Farms A and G, with none of the animals classified as a “high” shedder. In contrast, each of the infected farms had animals that were considered to be “high” shedders (2.3% to 20.0% of tested animals on the farms). In all cows with positive fecal tests, high shedders accounted for 6% to 38% in infected farms in this study. Previous studies have shown that the primary routes of MAP transmission within herds are from dam-to-daughter^26^, calf-to-calf^27^, as well as to naïve young animals and adults from other infected adults^5^. Based on simulation modeling of the transmission dynamics and persistence of MAP on commercial dairy farms, it has been suggested that high-shedding adults serve as the predominant contributor to the on-farm transmission of MAP^28^. Hence, it is tempting to speculate that animals within the NH group, although negative, were likely exposed to MAP-infected cows and may have been either early in infection (or were intermittent shedders that were missed during the sampling) or had recovered from a transient MAP infection and hence their fecal samples were found to be “negative”. Hence, serological reactivity to specific recombinant MAP proteins in the NH group is consistent with the hypothesis that these antigens may represent markers for early MAP infection or exposure, and it will be important for future investigations to rigorously test this hypothesis with well-designed studies of experimental and natural infections in cattle.

The current studies with MAP protein arrays identified 49 reactive antigens as potential serodiagnostic antigens for MAP infection. Importantly, the majority of these antigens were not recognized in previous investigations using MTB protein or smaller MAP protein arrays^9,16,21^, and have therefore greatly expanded the number of candidate antigens for the early (NH and F + E− groups) sero-diagnosis of MAP infections and highlight the fact that despite the similarity in protein sequences between MAP and MTB, the use of homologous proteins in studies such as these is critical.

Despite the fact that the majority of antigens identified in this study were previously unrecognized, several (*n* = 7) of these were, and showed increased sero-reactivity in infected animals in a manner consistent with our current observations. For instance, the secreted protein, MAP2609^9^, was identified as a significantly reactive antigen in the F + E+ group in our previous MTB protein microarray study^16^, and when used in a novel multiplex-bead based immunoassay format^14^, antibody activity to MAP2609 was recognized in both F + E− and F + E+ groups. Our current study found that MAP2609 is recognized in all three groups, suggesting that this antigen may be immunoreactive from early through late infection. Other antigens previously discovered to be immunoreactive or of potential diagnostic utility include MAP3527 (pep A) that was identified as reactive in the NH group in this study and also recognized in a previous study of naturally infected cattle^21^. Similarly, MAP2158, MAP2195, and MAP4147 that were observed to be immunoreactive in the F + E− group in the current study were also found to be immunoreactive in previous studies using previous immunoblotting and ELISA based approaches^12,15,29^. For instance, MAP2158 was recognized by sera from experimentally MAP-infected cows, but not by sera from *M. avium* subsp. *avium* and *M. bovis* infected animals, suggesting that this may represent an antigen specific to MAP^29^, and MAP4147 was recognized by sera from MAP-infected sheep in immunoblotting (though not in ELISA)^12^. In addition, MAP2942 and MAP3531 that were found to be reactive to sera from animals in the F + E+ group in the current study, were also recognized by sera from MAP infected cows in previous studies^9,21^. Of particular note, MAP2609 and MAP2942c, both secreted proteins, and recently identified as the most reactive antigens in the F + E+ group in the MTB protein array study^16^ and also further confirmed in our recent multiplex-bead based immunoassays^14^, were both reactive in the F + E+ group as well as in the F + E− group in both serum and milk samples, confirming their potential utility as diagnostic antigens. MAP3531, a secreted fibronectin binding protein (Antigen 85C) has also been evaluated in several previous studies where reactivity in the infected group was higher but not significantly different from the control^22^, however other studies have found it to be significantly (P < 0.05) more reactive with ELISA^21^ or immunoblotting^29^ based assays. Other candidate proteins such as MAP2705 and MAP2764 that were not identified as significantly reactive antigens in the current investigation, were also previously found to not differ in reactivity between groups in previous studies^29,30^.

It is noteworthy as well that several recombinant MAP proteins, including MAP0334, MAP0435c, MAP4056c, and MAP1204 + MAP1272c that have been previously recognized as reactive in infected animals were not seen to be differentially reactive in our current studies with MAP arrays^10–12,29^. Although p-values were < 0.05 in group comparison in this MAP array study, MAP0334 and MAP0435c were not identified as candidate antigens since their p-values were > 0.01 and they also had relatively low AUCs (<0.7). MAP1204 was recognized by sera from experimentally infected cows in the previous study^29^, but MAP1204 + MAP1272c was not included in candidate antigens since the odds ratio did not differ from the NL group although with a p-value for comparison of mean intensity <0.01.

The results showed that MAP4056c, a secreted protein, was previously identified from MAP culture filtrate and shown to be recognized by infected cows^11^, but was not included in our final list of candidates due to an overall low mean intensity even though it satisfied the selection criteria based on p-values, OR, and AUC, suggesting that further validation of the utility of this antigen for MAP diagnosis may be warranted. A total of 12 proteins, including MAP4056c, were similarly excluded from the final list of candidates since they had mean reactivities lower than 0.4 despite satisfying the inclusion criteria for p-value, OR, and AUC (Table S4: low intensity). One reason for this may be that the protein concentrations during array fabrication for each of these 12 proteins was lower than 0.1 mg/ml, and hence, may simply have been excluded for technical reasons since the data reveal a significant association between protein concentration and mean intensity for concentrations >0.1 mg/ml (p < 0.01; Fisher’s exact test and 2 × 2 contingency tables). Interestingly however, the data also show that low input protein concentration may not be the only reason for low reactivity since amongst 49 identified proteins (mean intensity >0.4 in at least in one group), 8 proteins with low protein concentration at printing (<0.05 mg/ml) were identified as highly reactive. For instance, even though MAP3531c had printing concentration <0.05 mg/ml, it had a high mean intensity in the F + E+ group (1.95; median 1.38) compared with low observed values in the NL, NH, and F + E− groups, suggesting that at least some antigens may exhibit strong immunoreactivity even at low concentrations. In general, however, the results highlight the potential confounding role of low input protein concentrations, and hence antigens that are excluded as not significantly reactive, might instead be “false negatives” and require additional investigations.

For further validation of the most promising antigen candidates, the 5 most reactive antigens were selected from each group, giving a total of 11 antigens including 3 secreted proteins, 1 lipoprotein, 1 conserved membrane protein and 6 predicted proteins, of which 5 are extracellular, 3 are membrane-bound, and 3 are cytoplasmic. The subcellular localization of bacterial antigens affects both accessibility by the immune response and antibody reactivity^20^. Although extracellular proteins only comprise about 0.5% of the proteome, they are more frequently recognized during the infection as observed in patients with active TB infection in the MTB proteome array study^20^. The results of our current studies showing that 5 out of 11 (45.5%) top candidates were extracellular proteins is consistent with the general observation that extra-cellular proteins are more likely to be identified as sero-reactive.

It is widely recognized that the next generation diagnostics for the detection of early MAP infection requires the identification of antigens that provide high sensitivity without compromising specificity. Although there is a slight reduction in the sensitivity in the heavily infected group (previously described with a sensitivity of 1 with the traditional ELISA) the use of the next generation of diagnostics will provide better management due to early detection of MAP-infection. This should improve the ability to detect animals in early stages of clinical infection and reduce within herd transmission or the introduction of animals that may be otherwise missed by traditional serological assays. When considered as individual antigens, the sensitivity of the top candidates was relatively low at a specificity cut-off of >0.950. Hence, we tested if antigen combinations might be used to increase the sensitivity to detect infection without compromising the specificity (Figs. 4B and 5B, 6B). The results show increasing levels of sensitivity with increased numbers of antigens in combination (sensitivity = 4-antigen>3-antigen>2-antigen combinations). However, increasing the antigen number to 5 did not provide a sensitivity benefit, suggesting that beyond a certain relatively small threshold number, no additional sensitivity gains might be expected without compromising specificity. Taken together, the results show that 3- or 4-antigen combinations displayed higher sensitivity without loss of specificity.

It appears that several of the candidate antigens identified in this study, particularly those that were differentially reactive in the NH and F + E− groups, may have potential utility for the early detection of MAP infection and may serve as the foundation for the next generation of well-defined serological tests for JD particularly when used in multiplex and high-throughput formats. One such commonly used high-throughput diagnostic technique with high sensitivity and specificity is the fluorescent bead-based multiplex immunoassay that has previously been successfully applied for serological diagnosis of human and animals infected by bacterial pathogens including *Borrelia burgdorferi, Chlamydia trachomatis, Streptococcus pneumoniae*, and *Haemophilus influenzae*^31–33^. This format has been recently adapted for MAP diagnosis^14^ based on antigens identified with MTB protein arrays^16^, and showed considerable promise with greater sensitivity for early detection of MAP-infected animals compared with conventional ELISAs. Interestingly, we show that antigens such as MAP2942c that are classified as seroreactive only in the F + E+ group with both MTB and MAP protein arrays, appear to show increased serological reactivity in both the F + E+ and F + E− groups when used in the multiplex-bead based assay format, suggesting that this assay format may provide additional advantages for the detection of early MAP infection in cattle.

The results of our studies are consistent with the observation that the nature of the immune response is dependent on the stage of infection, and that certain antigens that may be expressed and hence immunoreactive during early infection may or may not be immunoreactive at later stages. Hence, whether an animal is merely exposed (and becomes immunoreactive but not productively infected), infected – either latently or actively, diseased, or perhaps recovered from infection or disease, may be reflected in the constellation of antigens that are expressed and immunoreactive. Hence, it is axiomatic that assays be fit-for-purpose, and that assays that may be best suited to identify animals later during infection and thereby focused on reducing loss from disease, may indeed be different from assays that are best suited to identify animals early – before they become infectious – so as to drive towards elimination of infection in a farm or region.

Overall, the results with the protein microarrays are consistent with the hypothesis that specific antigens are likely differentially recognized by the immune system during the exposure, infection, disease continuum, and may serve as suitable biomarkers for the early detection of animals that are exposed and likely infected. Taken together, the results of our investigations suggest that MAP protein arrays have considerable utility in contributing to the discovery of novel antigens for the early detection of MAP infection in cattle, as well as provide a framework for future investigations to validate promising candidate antigens identified in this study, particularly in the NH and F + E− groups, with multiplex bead-based or similar high sensitivity immunoassays.

## Supplementary information

Supplemental Text

Supplementary Table 1

Supplementary Table 2

Supplementary Table 3

Supplementary Table 4
