# Supplementary material for: Identification of Sero-Diagnostic Antigens for the Early Diagnosis of Johne’s Disease using MAP Protein Microarrays
Source: Sci Rep. 2019 Nov 26;9:17573. doi: 10.1038/s41598-019-53973-x (PMC6879513; doi:10.1038/s41598-019-53973-x)
Supplement: Supplementary file 1 — Supplemental Text [file 41598_2019_53973_MOESM1_ESM.docx]

# **Identification of Sero-Diagnostic Antigens for the Early Diagnosis of Johne’s Disease using MAP Protein Microarrays**

Lingling Li^¶1, 2^, John P. Bannantine^¶3^, Joseph J. Campo^¶4^, Arlo Randall^4^, Yrjo T. Grohn^5^, Megan Schilling ^2,6^, Robab Katani^2, 7^, Jessica Radzio-Basu^2,7^, Laurel Easterling^2,6^, Vivek Kapur*^2,6,7^

**Supplemental Text**

Section A:

Analysis of the functional classification of these candidate antigens showed that 35% are involved in the cell wall and cell processes, 30% are not assigned any function, and the remaining 35% were involved in intermediate metabolism, lipid metabolism, information pathway, PE/PPE, regulatory, or virulence related functions. Based on predictions of subcellular localization ([Zhou et al., 2008](#_ENREF_35)) and proteomics-based studies with *Mycobacterium tuberculosis* ([Mawuenyega et al., 2005](#_ENREF_22); [Malen et al., 2007](#_ENREF_20); [Malen et al., 2010](#_ENREF_21)), the reactive antigens identified during our investigation represented membrane (53%), extracellular (14%), and cytoplasmic (33%) proteins, ratios that differ considerably from the prediction of over 75% of cytoplasmic proteins in the MAP genome ([Li et al., 2005](#_ENREF_18); [Zhou et al., 2008](#_ENREF_35))

Section B:

Interestingly, about half of the antigens (*n*=15) reactive in the NH group represent hypothetical proteins (S2 Table), with a majority (73.3%) of these predicted to be either membrane-associated or extracellular proteins

Section C:

Similar to that for the proteins reactive with the NH group, more than half of the reactive antigens (53.8%) are hypothetical, and the rest are involved in intermediary metabolism (23.1%), cell wall and cell process (19.2%), and information pathway (3.8%); and predicted subcellular locations suggest that half are membrane proteins, 42.3% cytoplasmic proteins, and 7.7% extracellular proteins.

Section D:

Similar to what was observed in the NL and F+E- groups, more than half of antigens identified in the F+E+ group were hypothetical (52.9%), while the others are predicted to be involved in cell wall and cellular processes, intermediary metabolism and lipid metabolism. Interestingly, nearly half (47.1%) of the reactive antigens in this group were predicted to be cytoplasmic, whereas the rest were predicted to be membrane (35.3%) or extracellular (17.6%) proteins.
